# For the American Journal of Dental Science

**Published:** 1840-07

**Authors:** Enoch Noyes

**Affiliations:** Dentist, Balt.


*For the American Journal of Dental Science.*

---

[ BY ENOCH NOYES—DENTIST, BALT. ]

---

Messrs. Editors,—

On the receipt of the first number of your highly valuable and useful journal, I took the liberty of addressing to you a short communication, congratulating you, and the profession generally, on the appearance of a publication so much and so long needed. I looked upon it as the precursor of a better condition of the profession, as the dawning of a new and bright epoch in its history. Thus far my expectations have been fully realized.

I have been disappointed with but one thing, and that is the irregularity in which its numbers have been issued. I can very readily imagine, however, that in starting the work, you have had many difficulties to contend with. Scattered as the members of the profession are over this vast country, it could not reasonably be expected that a subscription could have been obtained at once sufficiently large to defray the expenses of such a publication. Added to this, the deranged condition of the currency of the country, that which is good in one section not being available in another, may, I can well conceive, have occasioned unavoidable delays on the part of subscribers, in remitting their subscriptions. I rejoice, however, to learn that your list is steadily increasing, and cannot allow myself to doubt, that by the time the first twelve numbers shall have been issued, you will have obtained subscribers enough to justify you in the continuance of its publication.

A periodical of this kind has certainly long been needed, and now that one has been started, I hope means sufficient to sustain it will be furnished by the profession. Its influence, in connexion with that of the college that has recently been established in Baltimore for the education of gentlemen for the practice of this particular department of surgery, cannot be otherwise than salutary both to the profession and community at large. The former will be elevated by them to a place in the estimation of the latter, much higher than any which it has ever before occupied. I may be over sanguine, but I cannot but indulge the hope that, should the *Journal* and *College* be sustained, and I should regret exceedingly to think otherwise than that they will, the time is not far distant when, at least, most of those who shall be entrusted with the exercise of the duties of dentistry, will be men of science, men competent to practice in a manner creditable to themselves and beneficially to those by whom they may be employed. That the reverse of this to a very considerable extent at present is the fact, however painful and mortifying the acknowledgment, cannot be disguised.

In my former communication, I took occasion to advert to a species of mal-practice in our profession; that is, the filling of teeth with amalgams of mercury (quicksilver) and tin, or silver, usually denominated by those who use them, "metallic paste," and by the celebrated European empyrics—the *Crawcours,* assuming the more imposing appellation of

" *Royal Mineral Succedaneum*," and I am happy to find that in condemning this pernicious article, I am sustained by every scientific and experienced practitioner whose views upon the subject I have been able to learn. I should not again have alluded to the subject, had not Mr. Clark, one of your correspondents, proposed several enquiries in relation to it. That I shall be able to remove the doubts which his enquiries would seem to imply is more than I dare anticipate, for he tells us that the " dictum of no man, though grey in his profession, will be deemed sufficient." The inference to be deduced from this, is, that he is actually ignorant and chooses not to be taught, or in other words, that he will receive the testimony of no man, however much respect it may be entitled to. Anticipating the " imputation of ignorance," he contents himself with the philosophical reflection that " many a man has died a fool because he would not seek for knowledge, lest he expose his ignorance."

However well informed Mr. C. may be on other subjects, it must be evident to every one who has seen many teeth that had been filled with the article here alluded to, that he is really ignorant of the effects produced by it. Believing this to be the fact, I will proceed to answer the interrogatories proposed by him, which, whether or not satisfactory to him, will, I trust, be so to others.

To the question—" How does it appear that combinations of mercury, when employed as above stated, (refering to its use in the filling of teeth,) are necessarily destructive to the teeth ?" The answer to this is anticipated in part by the next question. " Does the external surface of the filling corrode, vitiate the saliva, and thus act externally on these organs ?" Most assuredly, and besides, by the evaporation of the mercury, the size of the filling is so lessened that the secretions of the mouth are frequently admitted between it and the walls of the tooth, and which by being retained there, induce a more rapid decomposition of the surrounding bone, than they would if the filling were not there, for the oxidized particles of this substance which mixes with them, increases their corrosive properties ; at least this would seem to be the case, from the fact, that teeth, after being filled with this amalgam, often decay faster than they did before.

The answer to the last question supersedes those which might be given to the two or three following : I would observe, however, that the mercury may " find its way into the stomach" but cannot " act through its influence on the organ," (meaning the tooth that had been filled with it. The gums, however, may be, for many have been salivated by having teeth stopped with it ; but that may have been attributable to its having been aken up by the absorbents of the mouth. It might, however, be produced in either way. I do not presume that it is ever taken up by the absorbents of the teeth, though I would not like to hazard the assertion that it was not. The solution of this question, however, is of no consequence to the point at issue. As it regards the *modus operandi*, that is a matter of no importance. The fact that bad effects do result from the use of the amalgam here alluded to, in filling teeth, ought to be sufficient to deter every dentist from employing it for that purpose. The answers however, which I have given to the interrogatories I believe to be correct, and in accordance with true philosophical principles.

There is one other question which it is proper to notice before dismissing the subject, and is thus propounded. "Whatever be the mode of injury, is it always speedily apparent, or is it slow, effecting imperceptibly, at first its destructive work ? And if the latter, about how long must we wait to have an experiment satisfactory ?" Much depends on the health of the mouth in other respects. In a healthy mouth, and where the cavity in the tooth is not very large, and has been previously well prepared, it may remain two or three years, before it becomes very apparent; but under opposite circumstances, it often manifests itself in two or three weeks. I extracted a tooth a few months since that had been filled with an amalgam of mercury and silver, about four or five weeks before, and the surface of the filling had become completely oxidized. I presented the tooth to the Baltimore editor of this journal, who can testify to the truth of the above statement, and who in a recent conversation informed me that he had extracted some fifteen or twenty teeth that had been filled with this article, and that he had removed this kind of filling from perhaps twice that number, and substituted gold in its place. He also stated that, ptyalism has been produced in several of the cases.

There is another fact noticed by the gentleman just refered to, in an editorial in the fourth number of the American Journal of Dental Science, and which ought forever to prevent the use of amalgams and alloys for filling teeth. It is that "metals when mixed have a much greater affinity for oxygen than when in a separate state." In another part of the same notice, he says, (refering to a comunication of his in a Baltimore paper, on the use of this amalgam for plugging teeth,) "we took occasion to advance the opinion that the rapid oxidation of the various '*metallic cements*' and '*pastes*' that are used for filling decayed teeth, was probably attributable to a galvanic action between the metals of which these '*amalgams*' are composed," and immediately goes on to add, "but whether this be true or not, the fact that they do oxidize, and that too very rapidly when placed in the mouth, should constitute an insuperable objection to their use." Now it is clear that it does oxidize, for the assertion made in the last part of this quotation, is evidently made from personal knowledge.

The New-York editor of the same journal, in a note to a supplementary treatise of Hunter, (and his opportunities for information on the subject have been abundant, and we may infer, therefore, that he speaks from knowledge,) thus alludes to the subject; "I regard" (says he) *cements*, (pastes,) fusible metals, amalgams, succedaneums, and all other substitutes for the above named metals," (gold, tin, or lead,) "as impositions on the public, never having seen a single operation, in which these substances were employed which would not have been more permanent if even lead, the poorest of the above named metals, had been used; because it is less subject to decomposition and oxidation, to say nothing of the poisonous qualities of the mercury which most of the others contain."

Pursuing the subject, the same gentleman adds,—"I have never known a perfect master of the art of stopping teeth, either to employ, or recommend the substances which I here condemn; and I believe the use of them is almost wholly confined to those persons who are unacquainted with this nice and difficult art."

The sentiments of the two gentlemen from whom I have quoted, touching this "amalgam" are no less severe than just, and emanating from such respectable sources, are at once entitled to general credence.

If the arguments that are here given in reply to the enquiries proposed by Mr. C., are not based upon facts sufficiently strong to convince him that "amalgams" of mercury and tin, or silver, are improper articles to stop teeth with, it is because he is determined not to be convinced.

If the explanation of the "*modus operandi*" of the "amalgam" in question on the teeth and mouth be not altogether satisfactory to him, the fact that its use is productive of injury to them, is nevertheless undeniable, and in further confirmation of which, I will subjoin the following cases. The names of the individuals I withold, though if any one questions the correctness of the statements, he can, by calling on me, obtain them.

Mrs. B., of Baltimore, about twelve months since, had three large cavities in her lower molar teeth filled with this article. Four or five days after the operation, her gums around the teeth that had been stopped, became sore, and in a few days extended to all her other teeth in her lower jaw. She at the same time complained of a mercurial taste. The secretions of her mouth were so abundant that she was kept almost constantly spitting; and they were much vitiated and very viscid. She at first did not know what this was attributable to. Imagining, however, they had been caused by the fillings in her teeth, she had them removed, and was at once relieved.

I know of several other persons that were affected in precisely the same way, by having teeth filled with this "amalgam," but the statement of the above, will suffice on this point.

Mr. L., of the District of Columbia, some years ago, had several teeth filled with gold, and a few months since, had two filled with what the dentist called a "*metallic paste*," but which was nothing more than an amalgam of mercury and silver, or tin. The mercury, in some way or other, got in contact with the gold fillings, and by uniting with them, caused them in a few weeks to crumble away and come out.

One other case, and I am done. The wife of the Rev. Mr. W., then of Virginia, but now I believe, of this city, had several artificial teeth inserted in her mouth, on gold plate five years ago, made fast to the adjoining natural ones by means of clasps. One of the teeth to which one of these had been attached, decayed, and while on a visit to this city, she was advised by a friend, to apply to an amalgam devotee, who was then making a great noise in the newspapers, about what he called a "metallic paste," which he used for filling teeth, and from the wonderful virtues ascribed to it, by its *advertisement fame*, she was induced to call on him and have her tooth filled with this substance. The result was, the mercury of the "*paste*" got on the gold clasp, soon extended to the plate, and in a very short time, caused both to crumble to pieces.

Of the injurious results produced by the use of that despicable compound, the amalgam of mercury, hundreds of examples might be adduced, but enough has already been said to convince even the most skeptical; I therefore, take leave of the subject for the present.

